# First-time diagnosis and referral practices for individuals with CKD by primary care physicians: a study of electronic medical records across multiple clinics in Japan

**DOI:** 10.1007/s10157-025-02695-8

**Published:** 2025-05-16

**Authors:** Haruhito A. Uchida, Jun Wada, Yuji Nagao, Katsuhito Ihara

**Affiliations:** 1 Department of Nephrology, Rheumatology, Endocrinology and Metabolism, Faculty of Medicine, Dentistry and Pharmaceutical Sciences, Okayama University, 2-5-1 Shikata-Cho, Kita-Ku, Okayama, 700-8558 Japan; 2 Department of Chronic Kidney Disease and Cardiovascular Disease, Faculty of Medicine, Dentistry, and Pharmaceutical Science, Okayama University, 2-5-1 Shikata-Cho, Kita-Ku, Okayama, Japan; 3 Medicine Division, Nippon Boehringer Ingelheim Co., Ltd., 2-1-1 Osaki, Shinagawa-Ku, Tokyo, 141-6017 Japan

**Keywords:** Chronic kidney disease, Electronic medical records, Japan, Primary care physician, Disease code

## Abstract

**Background:**

Chronic kidney disease (CKD) is a major public health burden in Japan. Japanese primary care physicians (PCPs) are expected to play an important role in the early diagnosis and management of CKD, but comprehensive data on their role are limited.

**Methods:**

This observational study examined data from individuals who underwent tests for CKD diagnosis between January 2017 and September 2023 in the Japan Medical Data Survey (JAMDAS) database of primary care clinics in Japan. The primary outcome was the proportion of individuals with CKD without the registration of a CKD-related disease code. Time to CKD diagnosis and referral were also assessed.

**Results:**

Among 1,188,543 eligible individuals who underwent kidney-related laboratory tests, 183,473 (15.4%) met CKD diagnosis criteria according to the Japanese Clinical Practice Guideline for CKD. The mean (± SD) age was 77.4 ± 11.0 years, 57.1% were female, and 71.8% had CKD stage 3a. Over 98% of individuals who met CKD diagnosis criteria did not receive an insurance diagnosis code within 90 days after meeting the criteria. Among referrable individuals, 89.7% did not receive a referral within 90 days of meeting the referral criteria.

**Conclusion:**

These results suggest CKD may be underdiagnosed and under-referred in Japanese clinics. Measures should be taken to increase detection and diagnosis according to the Japanese Clinical Practice Guideline for CKD.

**Supplementary Information:**

The online version contains supplementary material available at 10.1007/s10157-025-02695-8.

## Introduction

Chronic kidney disease (CKD) is a global public health burden, with its prevalence increasing in Asia, including Japan [1, 2]. The prevalence of CKD in Japan is ~ 14.6% [3], and the socioeconomic burden of dialysis in patients with end-stage kidney disease (ESKD) is a critical issue [1, 4–6]. CKD is linked to increased risk of cardiovascular (CV) and metabolic disorders [7], further worsening the burden of disease [8].

The Kidney Disease: Improving Global Outcomes (KDIGO) guidelines recommend early intervention and appropriate management of CKD [9]. Early detection of CKD and referral to a nephrologist could slow the progression of CKD and associated complications [10–12]. Regular assessment of estimated glomerular filtration rate (eGFR) values using serum creatinine (SCr) levels and urinalysis can identify patients with early-stage CKD [13].

Despite having a decline in kidney function, patients with early-stage CKD often fail to seek medical care because they are primarily asymptomatic [14]. CKD is frequently diagnosed at later stages, delaying initiation of effective interventions [15]. Even when patients with declining kidney function visit a hospital or clinic, physicians may overlook early signs of CKD without evaluating eGFR or urinalysis. This lack of early diagnosis, prognostic assessment, and management puts patients at greater risk of disease progression and complications, including ESKD and CV events [15].

Previous research has shown a low rate of early-stage CKD diagnosis in several countries, including Japan [15, 16]. Those with CKD-related diagnosis codes were more likely to receive CKD treatment and present with comorbidities. Patients were more likely to be diagnosed by nephrologists and diabetologists, suggesting a better understanding of CKD treatment in these background specialties, increasing the chances of CKD diagnosis. However, in Japan, the ratio of nephrologists to CKD patients is alarmingly low, with only 6201 nephrologists (July 2023 [17]) for 14.8 million CKD patients (2015 [3]).

Most CKD patients are classified as stage G3a and treated by primary care physicians (PCPs) [16, 18], with Japanese PCPs expected to play a key role in managing CKD patients as their initial point of contact. The Japanese Clinical Practice Guideline for CKD (hereafter referred to as “Japanese CKD guidelines”) advises PCPs to refer CKD patients at stage G3b or worse to nephrologists to slow progression of CKD and associated complications [19, 20]. However, comprehensive data on CKD diagnosis and referrals to other hospitals among patients attending primary care clinics (PCCs) are scarce. In this retrospective study, we used the Japan Medical Data Survey (JAMDAS) database to determine the proportion of individuals with CKD without a disease code registration, time to diagnosis, and referral practices. Our results will further our understanding of CKD management in PCCs in Japanese clinical practice, and prompt discussions on how to improve CKD management to delay disease progression.

## Methods

### Study design and data source

**Fig. 1:**
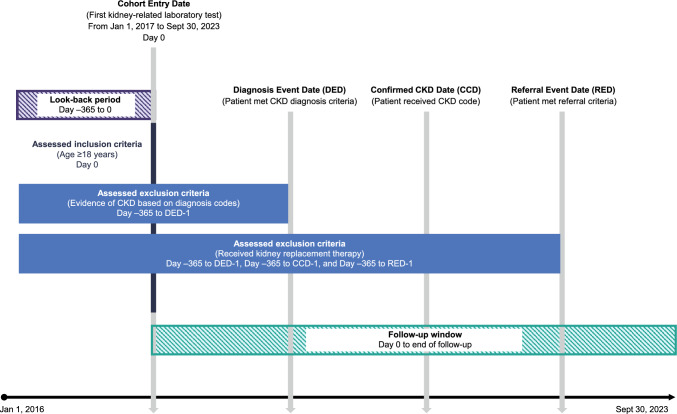
Study design. *CCD* confirmed CKD date, *CKD* chronic kidney disease, *DED* diagnosis event date, *RED* referral event date, *y* year

This was an observational study using real-world data from the JAMDAS database (M3, Inc., Japan) [21, 22]. The study design is shown in Fig. 1. The database includes clinical practice information from ~ 31 million individuals from 4700 PCCs in Japan. It includes all outpatient visits to the clinics and individual-level information on diagnosis or disease names for health insurance claims, prescriptions, medical practices provided, vital signs, laboratory data, and clinic location. Individual and clinic identifiers are available in an anonymized form. The study protocol received institutional review board (IRB) approval from the Takahashi Clinic Ethics Committee on December 12, 2023 (IRB approval number: LNW00205).

### Study population

Inclusion criteria included being ≥ 18 years with ≥ 1 kidney-related laboratory test (Online Resource 1), regardless of values, between January 1, 2017 and September 30, 2023.

To investigate patients newly identified as eligible for CKD diagnosis and referral in clinical practice we sought to exclude patients who had already been diagnosed with CKD or had reached ESKD at baseline. As some patients can meet referral criteria to specialists with only one laboratory value meeting diagnosis criteria, we needed to exclude those with a CKD-related diagnosis code before the first instance of laboratory values meeting diagnosis criteria as well as those with a CKD-related diagnosis code between the first and second instances of laboratory values meeting diagnosis criteria. Accordingly, exclusion criteria were the presence of CKD-related diagnosis codes (Online Resource 2), or undergoing kidney replacement therapy (Online Resource 3) before the date of the first: 1) kidney-related laboratory test; 2) laboratory values meeting diagnosis criteria according to Japanese CKD guidelines (Online Resource 4) at least once; or 3) laboratory values meeting the diagnosis criteria according to Japanese CKD guidelines at least twice consecutively with an interval of ≥ 3 months. Neither were individuals undergoing kidney replacement therapy on or before the date of 1) the first laboratory values meeting the referral criteria according to Japanese CKD guidelines (Online Resource 5); or 2) any evidence of CKD diagnosis based on CKD-related diagnosis codes, included in the study. Individuals without registration in the JAMDAS database after the date of the first kidney-related laboratory test were excluded. Diagnosis and referral criteria for CKD were based on eGFR, which was calculated using the Japanese eGFR equation from the Japanese Society of Nephrology: eGFR (mL/min/1.73 m^2^) = 194 × SCr value (mg/dL)^−1.094^ × age (y)^−0.287^ (× 0.739 [if female]) [23].

Among individuals who met the eligibility criteria (hereinafter “eligible individuals”), this analysis focused on individuals who met the CKD diagnosis criteria, individuals who met the CKD referral criteria (hereinafter “referrable individuals”), and individuals with a CKD diagnosis (Fig. 1).

### Outcomes

The primary outcome was the proportion of individuals with CKD without a registration of a CKD-related disease code, using the *International Classification of Diseases, Tenth Revision (ICD-10)* (Online Resource 2). CKD was classified based on cause, glomerular filtration rate category, and albuminuria category (CGA). The periods from the date of laboratory values meeting the CKD diagnosis criteria to the date of registration of a CKD-related disease code, and the proportion of individuals referred to other medical institutions were also evaluated. Evidence of referral to other institutions was evaluated on the presence of a referral document to another medical institution (Online Resource 6), regardless of the purpose.

Subgroup analyses were performed for: CKD severity category at baseline (Online Resource 7), year the laboratory values indicated CKD and the need for referral to a nephrologist, clinic location, age, and the presence of urinary tests (Online Resource 8). Codes for medical history/comorbidities and medications are listed in Online Resources 9 and 10, respectively.

### Statistical analysis

Summary statistics were provided for continuous variables, and frequencies were recorded for categorical variables. The primary outcome was the proportion of individuals with CKD without registration of a CKD-related disease code, with a 95% confidence interval (CI) calculated using the Clopper-Pearson method. A sensitivity analysis of the primary outcome was performed based on a broader definition for CKD-related diagnosis codes (Online Resource 11). The time-to-event distribution was analyzed using the Kaplan–Meier method, with the median survival time and 95% CI based on Greenwood’s formula. All data were analyzed using SAS version 9.4 (SAS Institute Inc., Cary, NC, USA).

## Results

### Study population

**Fig. 2:**
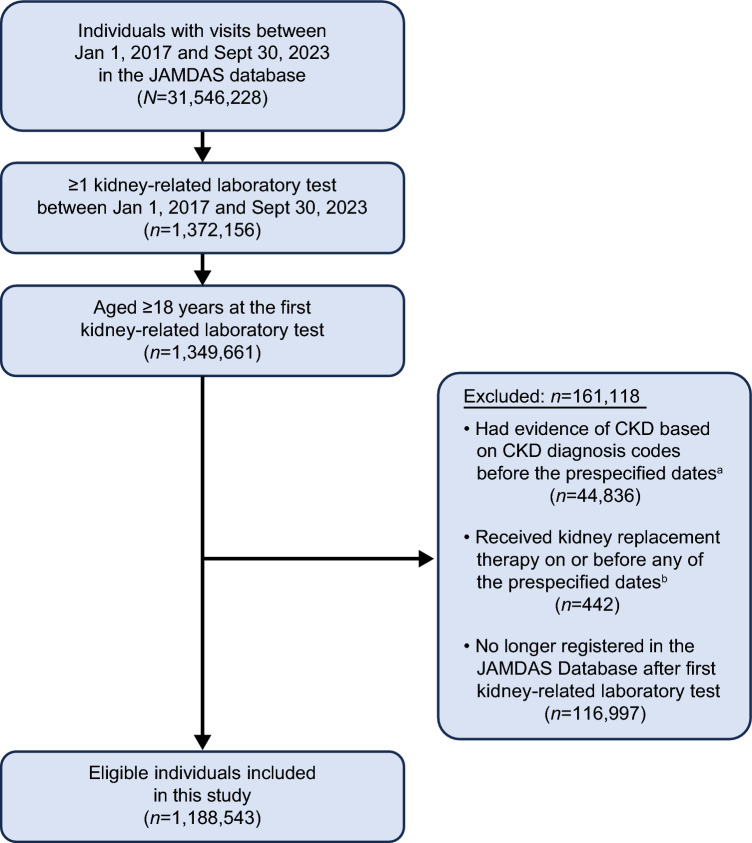
Study population. *CKD* chronic kidney disease, *JAMDAS* Japan Medical Data Survey. ^a^Prespecified dates were: 1) date of the first kidney-related laboratory test; 2) date of the first laboratory values meeting the diagnosis criteria according to Japanese CKD guidelines at least once; 3) date of the first laboratory values meeting the diagnosis criteria according to Japanese CKD guidelines at least twice consecutively with an interval of at least 3 months. ^b^Prespecified dates were: 1) date of the first kidney-related laboratory test; 2) date of the first laboratory values meeting the diagnosis criteria according to Japanese CKD guidelines at least once; 3) date of the first laboratory values meeting the diagnosis criteria according to Japanese CKD guidelines at least twice consecutively with an interval of at least 3 months; 4) date of the first laboratory values meeting the referral criteria according to Japanese CKD guidelines; 5) date of any evidence of CKD diagnosis based on CKD-related disease codes

Among 1,188,543 eligible individuals who underwent kidney-related laboratory tests (Fig. 2), 183,473 (15.4%) met diagnosis criteria according to Japanese CKD guidelines. Baseline characteristics of eligible individuals who met the diagnosis criteria are summarized in Table 1. We divided those who met the CKD diagnosis criteria into two groups: those with a CKD-related disease code and those without (Table 1). The breakdown of CKD diagnosis criteria in individuals who met CKD diagnosis criteria are provided in Online Resource 13. Baseline characteristics were categorized by clinic location and CGA category (Online Resource 14 and Online Resource 15, respectively).

**Table 1:** Demographics and characteristics for all individuals eligible for the study and those who met the CKD diagnosis criteria

	Individuals eligible for the study (*n* = 1,188,543)	Individuals who met the CKD diagnosis criteria			
		Total (*n* = 183,473)	With CKD code^a^ (*n* = 2853)	Without CKD code (*n* = 180,620)	*p*-value
Age, years	61.5 ± 18.7	77.4 ± 11.0	76.2 ± 12.3	77.4 ± 11.0	< 0.01
Female	672,511 (56.6)	104,846 (57.1)	1293 (45.3)	103,553 (57.3)	< 0.01
BMI, kg/m^2^	*n* = 169,310; 23.5 ± 4.0	*n* = 40,665; 23.7 ± 3.7	*n* = 832; 24.2 ± 4.1	*n* = 39,833; 23.6 ± 3.7	< 0.01
< 18.5	14,353 (1.2)	2771 (1.5)	57 (2.0)	2714 (1.5)	< 0.01
≥ 18.5–25	101,476 (8.5)	24,434 (13.3)	446 (15.6)	23,988 (13.3)	
≥ 25–30	41,777 (3.5)	11,127 (6.1)	257 (9.0)	10,870 (6.0)	
≥ 30	11,704 (1.0)	2333 (1.3)	72 (2.5)	2261 (1.3)	
Data not available	1,019,233 (85.6)	142,808 (77.8)	2,021 (70.8)	140,787 (77.9)	
Systolic blood pressure, mmHg	*n* = 436,358; 132.9 ± 20.9	*n* = 84,312; 133.2 ± 18.7	*n* = 1,646; 133.7 ± 20.3	*n* = 82,666; 133.2 ± 18.7	0.29
Diastolic blood pressure, mmHg	*n* = 436,230; 77.1 ± 13.5	*n* = 84,291; 73.6 ± 12.1	*n* = 1,646; 73.1 ± 13.2	*n* = 82,645; 73.6 ± 12.1	0.06
HbA1c, %	*n* = 940,898; 5.8 ± 0.9	*n* = 124,954; 6.0 ± 0.8	*n* = 1,692; 6.0 ± 0.9	*n* = 123,262; 6.0 ± 0.8	< 0.01
Hb, g/dL	*n* = 956,888; 13.7 ± 1.7	*n* = 145,717; 13.0 ± 1.7	*n* = 2231; 12.7 ± 2.1	*n* = 143,486; 13.0 ± 1.7	< 0.01
Serum albumin, g/dL	*n* = 382,715; 4.3 ± 0.4	*n* = 62,215; 4.1 ± 0.4	*n* = 1,147; 4.0 ± 0.5	*n* = 61,068; 4.1 ± 0.4	< 0.01
Serum calcium, mg/dL	*n* = 154,939; 9.1 ± 0.4	*n* = 26,722; 9.1 ± 0.5	*n* = 512; 9.1 ± 0.6	*n* = 26,210; 9.1 ± 0.5	0.28
CKD stage by eGFR, mL/min/1.73 m^2^	*n* = 1,134,840; 74.6 ± 20.3	*n* = 179,426; 49.8 ± 9.9	*n* = 2791; 44.5 ± 14.3	*n* = 176,635; 49.9 ± 9.8	< 0.01
G1 (≥ 90)	218,163 (18.4)	546 (0.3)	30 (1.1)	516 (0.3)	< 0.01
G2 (≥ 60–90)	671,284 (56.5)	2784 (1.5)	97 (3.4)	2687 (1.5)	
G3a (≥ 45–60)	186,031 (15.7)	131,810 (71.8)	1337 (46.9)	130,473 (72.2)	
G3b (≥ 30–45)	48,123 (4.0)	36,682 (20.0)	907 (31.8)	35,775 (19.8)	
G4 (≥ 15–30)	9559 (0.8)	6912 (3.8)	370 (13.0)	6542 (3.6)	
G5 (< 15)	1680 (0.1)	692 (0.4)	50 (1.8)	642 (0.4)	
Data not available	53,703 (4.5)	4047 (2.2)	62 (2.2)	3985 (2.2)	
UPCR, g/gCr Median (Q1, Q3)	*n* = 8453; 0.09 (0.05, 0.21)	*n* = 1897; 0.21 (0.09, 0.52)	*n* = 144; 0.16 (0.06, 0.56)	*n* = 1,753; 0.22 (0.10, 0.52)	0.93
A1 (< 0.15)	5613 (0.5)	665 (0.4)	67 (2.3)	598 (0.3)	< 0.01
A2 (≥ 0.15–0.50)	1784 (0.2)	734 (0.4)	40 (1.4)	694 (0.4)	
A3 (≥ 0.15)	1056 (0.1)	498 (0.3)	37 (1.3)	461 (0.3)	
Data not available	1,180,090 (99.3)	181,576 (99.0)	2709 (95.0)	178,867 (99.0)	
UACR, mg/gCr Median (Q1, Q3)	*n* = 10,274; 14.0 (6.9, 38.8)	*n* = 2252; 39.2 (14.3, 89.9)	*n* = 306; 55.2 (20.2, 179.0)	*n* = 1946; 38.0 (13.6, 80.7)	< 0.01
A1 (< 30)	7220 (0.6)	860 (0.5)	97 (3.4)	763 (0.4)	< 0.01
A2 (≥ 30–300)	2591 (0.2)	1,212 (0.7)	155 (5.4)	1057 (0.6)	
A3 (≥ 300)	463 (0.0)	180 (0.1)	54 (1.9)	126 (0.1)	
Data not available	1,178,269 (99.1)	181,221 (98.8)	2547 (89.3)	178,674 (98.9)	
Urinalysis (qualitative)	*n* = 65,290	*n* = 10,043	*n* = 219	*n* = 9,824	
A1 (−)	50,979 (4.3)	4301 (2.3)	86 (3.0)	4215 (2.3)	0.06
A2 (±)	7122 (0.6)	2797 (1.5)	53 (1.9)	2744 (1.5)	
A3 (+ ~ 4 +)	7189 (0.6)	2945 (1.6)	80 (2.8)	2865 (1.6)	
Data not available	1,123,253 (94.5)	173,430 (94.5)	2,634 (92.3)	170,796 (94.6)	
Comorbidities					
Type 1 diabetes	1857 (0.2)	316 (0.2)	9 (0.3)	307 (0.2)	0.06
Type 2 diabetes	292,103 (24.6)	74,037 (40.4)	1490 (52.2)	72,547 (40.2)	< 0.01
Hypertension	425,462 (35.8)	126,054 (68.7)	2128 (74.6)	123,926 (68.6)	< 0.01
Dyslipidemia	382,163 (32.2)	103,516 (56.4)	1,673 (58.6)	101,843 (56.4)	0.02
Heart failure	104,777 (8.8)	44,719 (24.4)	877 (30.7)	43,842 (24.3)	< 0.01
Chronic artery disease	78,334 (6.6)	30,723 (16.7)	523 (18.3)	30,200 (16.7)	0.02
Myocardial infarction	11,566 (1.0)	4958 (2.7)	104 (3.6)	4854 (2.7)	< 0.01
Angina pectoris	65,576 (5.5)	25,738 (14.0)	398 (14.0)	25,340 (14.0)	0.90
Stroke	32,256 (2.7)	12,376 (6.7)	145 (5.1)	12,231 (6.8)	< 0.01
Peripheral vascular disease	42,243 (3.6)	14,589 (8.0)	232 (8.1)	14,357 (7.9)	0.72
Atrial fibrillation	33,464 (2.8)	15,766 (8.6)	299 (10.5)	15,467 (8.6)	< 0.01
Medications					
Active vitamin D drug	22,148 (1.9)	12,948 (7.1)	171 (6.0)	12,777 (7.1)	0.03
* RAS* inhibitor	150,748 (12.7)	71,443 (38.9)	1367 (47.9)	70,076 (38.8)	< 0.01
Beta-blocker	37,780 (3.2)	20,680 (11.3)	401 (14.1)	20,279 (11.2)	< 0.01
Calcium channel blocker	174,528 (14.7)	72,637 (39.6)	1,275 (44.7)	71,362 (39.5)	< 0.01
Diuretic	38,088 (3.2)	26,095 (14.2)	588 (20.6)	25,507 (14.1)	< 0.01
Statin	130,271 (11.0)	53,723 (29.3)	849 (29.8)	52,874 (29.3)	0.45
DPP-4 inhibitor	52,356 (4.4)	23,355 (12.7)	661 (23.2)	22,694 (12.6)	< 0.01
SGLT2 inhibitor	33,952 (2.9)	15,757 (8.6)	463 (16.2)	15,294 (8.5)	< 0.01
GLP-1 RA	2159 (0.2)	1248 (0.7)	61 (2.1)	1187 (0.7)	< 0.01
Biguanide	25,421 (2.1)	10,196 (5.6)	318 (11.1)	9878 (5.5)	< 0.01
Sulfonylurea	11,026 (0.9)	4956 (2.7)	126 (4.4)	4830 (2.7)	< 0.01
Other oral glucose-lowering drug	12,596 (1.1)	5929 (3.2)	165 (5.8)	5764 (3.2)	< 0.01
Insulin	4169 (0.4)	2543 (1.4)	119 (4.2)	2424 (1.3)	< 0.01

Data are (%) or mean ± SD unless otherwise indicated. The evaluation periods for the covariates are described in Online Resource 12

*BMI* body mass index, *CKD* chronic kidney disease, *DPP-4* dipeptidyl peptidase-4, *eGFR* estimated glomerular filtration rate, *GLP-1 RA* glucagon-like peptide-1 receptor agonist, *Hb* hemoglobin, *HbA1c* glycated hemoglobin, *Q1* first quartile, *Q3* third quartile, *RAS* renin-angiotensin system, *SD* standard deviation, *SGLT2* sodium-glucose cotransporter-2, *UACR* urine albumin-to-creatinine ratio; *UPCR* urine protein-to-creatinine ratio

^a^Presence of CKD-related disease codes based on the *ICD-10*

The mean (± SD) age of individuals who met the CKD diagnosis criteria was 77.4 ± 11.0 years, and 57.1% were female. A limited number of individuals underwent urine tests (5.5%). Most individuals who met the diagnosis criteria were categorized with CKD stage G3a based on eGFR values (71.8%; 131,810/183,473). Additionally, 40.4% of individuals who met diagnosis criteria (74,037/183,473) had type 2 diabetes (T2D), 68.7% (126,054/183,473) had hypertension, and 56.4% (103,516/183,473) had dyslipidemia (Table 1).

When analyzed based on CKD-related disease codes within 90 days of meeting CKD diagnosis criteria, the distribution of CKD stages was different. The percentages were lower in the early stages of CKD and higher in the later stages for those with CKD-related disease codes (G3a: 46.9%, G3b: 31.8% and G4: 13.0%) compared with those without diagnosis codes (G3a: 72.2%, G3b: 19.8%, G4: 3.6%) (Table 1). Individuals with CKD-related disease codes within 90 days had a higher prevalence of cardio-renal-metabolic disease (heart failure [30.7%], hypertension [74.6%], and T2D [52.2%]) than those without disease codes (24.3, 68.6, and 40.2%, respectively) (Table 1).

Individuals with CKD-related disease codes at any time during the follow-up period (Online Resource 16) were younger, with higher rates of stages G1/G2 and T2D, and lower rates of heart failure and hypertension than those with disease codes within 90 days of meeting the diagnosis criteria (Table 1). Individuals who met referral criteria (Online Resource 17) were older with lower eGFR, undergoing urinalysis more frequently than those who met the diagnosis criteria (Table 1). Individuals who met the referral criteria by CGA category and clinic location are shown in Online Resource 18 and Online Resource 19, respectively.

### Unregistered disease code rates

**Fig. 3:**
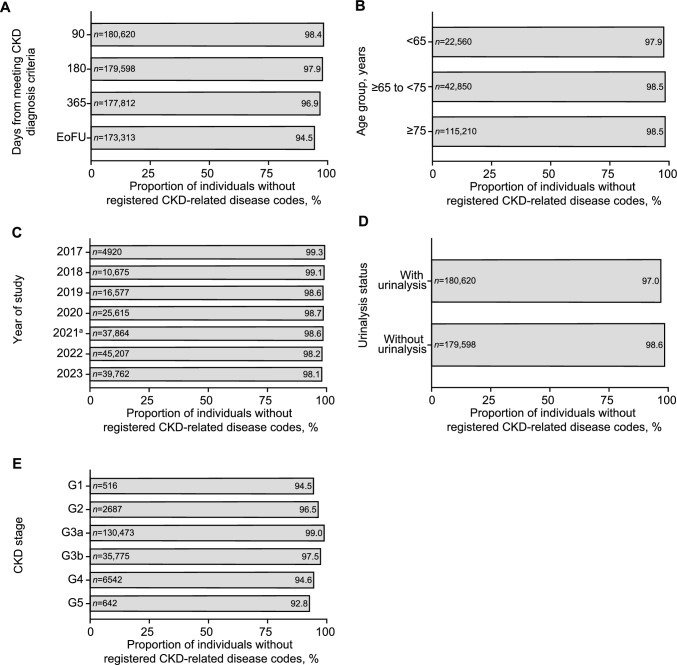
Proportion of individuals without registered CKD-related disease codes: overall (**a**), and stratified by age group (**b**), year (**c**), urinalysis status (**d**), and CKD stage (**e**) in individuals who met the CKD diagnosis criteria. ^a^The year the first medication (an SGLT2 inhibitor) with an indication for CKD was approved. *CKD* chronic kidney disease, *EoFU* end of follow-up, *SGLT2* sodium-glucose co-transporter-2

The rate of individuals without diagnosis codes was 98.4% within 90 days after meeting diagnosis criteria for CKD (Fig. 3a). The rate was similar among individuals with ≥ 1 visit after meeting diagnosis criteria for CKD (98.4%). The rates were similar at 180 and 365 days after meeting diagnosis criteria, with 97.9 and 96.9% of individuals without diagnosis codes, respectively (Fig. 3a), and similar among different age groups (Fig. 3b). Rates declined as the study progressed (Fig. 3c) and in individuals with versus without urinalysis (Fig. 3d). Rates of individuals without diagnosis codes were higher among earlier stages of CKD versus later stages (Fig. 3e). Rates of individuals without diagnosis codes appeared similar regardless of clinic location (Online Resource 20).

The breakdown of CKD-related diagnosis codes in individuals with codes within 90 days of meeting the CKD diagnosis criteria, and the sensitivity analysis, are shown in Online Resources 21 and 22, respectively. The rate of individuals without a CKD-related disease code using a broader definition for CKD-related disease, and for individuals who met CKD diagnosis criteria at least once, were similar to those of the primary outcome (Online Resource 23).

### Time gap between CKD tests and registration of CKD-related disease codes

**Fig. 4:**
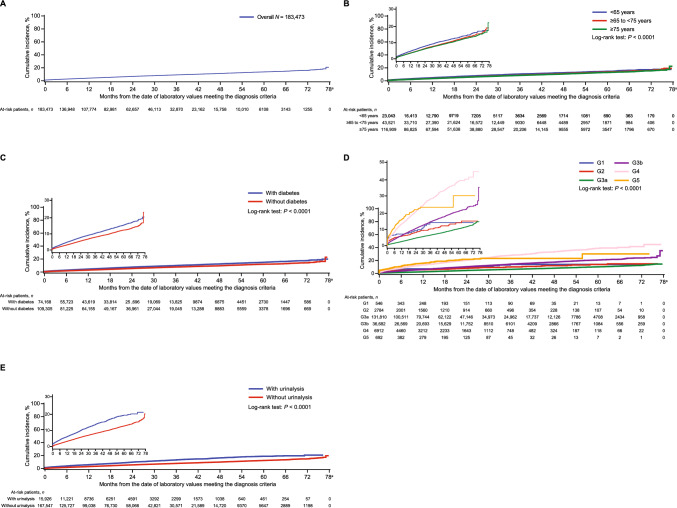
Time gaps from the date of laboratory values meeting the CKD diagnosis criteria to that of receiving CKD-related disease codes using Kaplan–Meier curves for all individuals who met the diagnosis criteria (**a**), and stratified by age group (**b**), diabetes status (**c**), CKD stage (**d**), and urinalysis status (**e**). Each inset shows the same data on an enlarged y-axis. *CKD* chronic kidney disease. ^a^All individuals were censored at 78 weeks, as this was the maximum follow-up period. This period of 78 weeks was determined by a combination of the data extraction period and the date when laboratory values met the diagnosis criteria

Figure 4a shows the time intervals from meeting the CKD diagnosis criteria to receiving CKD-related disease codes for all individuals who met the diagnosis criteria. The time to receiving CKD-related disease codes was delayed with increasing age (Fig. 4b). The registration rate of CKD-related disease codes was higher among individuals with T2D versus without (Fig. 4c). Time to receiving CKD-related disease codes was shorter in individuals with later CKD stages (G4 and G5) than with earlier stages (Fig. 4d), and registration rates of CKD-related disease codes were lower among individuals with earlier CKD stages (G1, G2, and G3a). Additionally, individuals with urinalysis received a diagnosis code sooner than those without (Fig. 4e). When stratified by CKD stage, patients with CKD stages G3a to G4 who underwent urinalysis tests had a higher rate of CKD-related disease code registration and a quicker time to disease code registration that those who did not (Online Resource 24).

### Non-referral rates

**Fig. 5:**
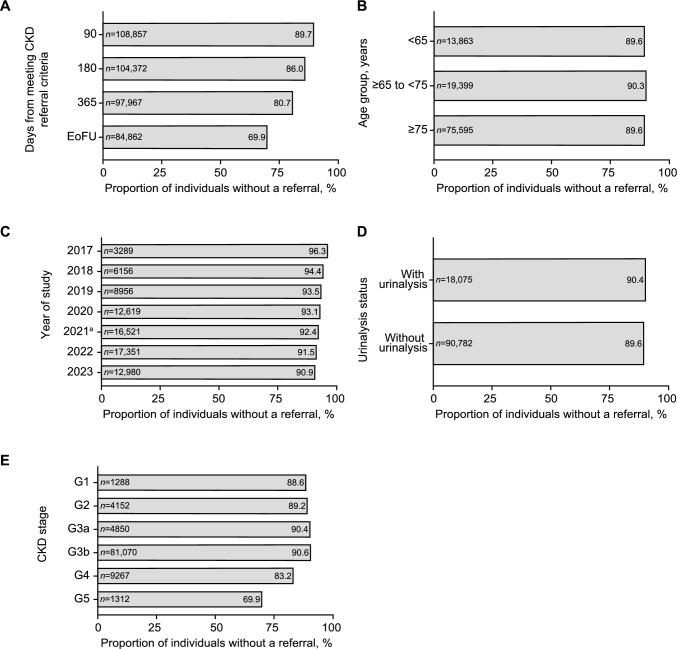
Proportion of non-referred individuals: overall (**a**) and stratified by age group (**b**), year (**c**), urinalysis status (**d**), and CKD stage (**e**) in individuals who met the CKD referral criteria. ^a^The year the first medication (an SGLT2 inhibitor) with an indication for CKD was approved. *CKD* chronic kidney disease, *EoFU* end of follow-up, *SGLT2* sodium-glucose co-transporter-2

Among individuals who met referral criteria, 89.7% were not referred within 90 days of meeting criteria (Fig. 5a). This proportion decreased to 86.0% at 180 days and 80.7% at 365 days. The proportion of non-referred individuals was similar across age groups (Fig. 5b), and urinalysis status (Fig. 5d), but declined gradually as the study progressed (Fig. 5c). Individuals in the later stages of CKD were more likely to be referred than those in the earlier stages (Fig. 5e). The proportion of non-referred individuals by clinic location is shown in Online Resource 20.

## Discussion

Here, real-world data were used to investigate diagnosis and referral patterns in individuals with CKD among PCCs in Japan. In individuals who met the diagnosis criteria according to Japanese CKD guidelines, rates of individuals without registered CKD-related disease codes were high, with > 98% of individuals not receiving a diagnosis within 90 days of meeting diagnosis criteria. Individuals were more likely to receive an earlier diagnosis in the later disease stages (G4 or G5) or if they had urinalysis. The rate of referral to a specialist was low, with 89.7% of referrable individuals not receiving a referral within 90 days of meeting referral criteria. Rates of unregistered disease codes and non-referred individuals decreased slightly over time. However, even after the first medication for CKD, a sodium-glucose co-transporter-2 inhibitor, was approved in 2021, diagnosis and referral rates were still low in the subsequent 2 years.

Various factors could account for the high rate of unregistered disease codes cases in early-stage CKD. In elderly patients, it may be assumed by physicians that eGFR values indicating stage 3 CKD are due to age-related decline in kidney function. However, even among older adults with a lower risk of kidney failure, stage 3 CKD is associated with an increased risk of mortality, CV events, and acute kidney injury [24], with additional complications of frailty and sarcopenia [25]. As such, KDIGO guidelines advocate using a single threshold value to define CKD across all age subgroups, aligning with criteria for other chronic non-communicable diseases. It is crucial for physicians not to underestimate the impact of CKD in elderly patients, and to promptly initiate guideline-appropriate management, adapted according to age, frailty, comedications, and comorbidities.

Infrequent use of urinary tests in Japan [26] may explain the low rate of early registration of CKD-related disease codes. In this study, qualitative urinalysis data was not available for over 94% of individuals included in the study, and quantitative analysis was similarly low. Since the early stages of CKD are typically asymptomatic, kidney function-related examinations are not commonly conducted in clinical practice [27]. This is particularly true for urinary tests, which require an additional order for examination. Japanese CKD guidelines emphasize that urinary tests are essential for diagnosing and assessing the severity of CKD [19]. It is worth noting that individuals with urinalysis data received disease codes sooner and had a higher rate of disease code registration than those who did not, which is consistent with previous literature [28]. Considering that CKD detection and treatment can reduce the risk of kidney failure by slowing CKD progression and the risk of CV disease [9], an accurate, timely diagnosis of CKD could result in effective care for patients with CKD.

It should be noted that in this study, the disease code registration rate for CKD does not necessarily reflect whether clinicians recognize CKD; however, this may be an indicator of clinicians recognizing the importance of CKD, and that registering a CKD-related disease code may signify the recognition of the condition as an important disease. Increased registration of CKD diagnosis codes could contribute to achieving a common understanding of CKD with high certainty among physicians, patients, and paramedical staff. We recommend diagnosing CKD and/or referring to nephrologists as early as possible, as early diagnosis and referral are associated with improved prognosis [10–12]. Collaboration between PCPs and nephrologists should be facilitated, as studies have reported that significantly higher rates of referral and co-treatment slow progression of CKD [29, 30].

A primary strength of this study is the large population of individuals examined, which allowed for a realistic description of CKD diagnosis and referral practices in Japan. The JAMDAS database contains individual-level information collected from ~ 4700 PCCs, making up ~ 5% of PCCs in Japan, and includes longitudinal data over time. To the best of our knowledge, this is the most extensive database in Japan, encompassing both claims data and laboratory results. We did not observe an association between the number of nephrologists in each prefecture and the unregistered disease code rate or non-referral rate for patients with CKD (data not shown). More studies are needed to elucidate the relationship between medical access and CKD diagnoses and referrals, and how this affects CKD prognosis. Previously, there has been limited research focusing on practices for managing CKD in PCCs. Secondly, this study does not include patients from core hospitals (or “big units”). These hospitals typically have multiple specialists, specialized outpatient clinics, and can perform imaging diagnostics and detailed medical examinations that could lead to faster diagnoses. However, the primary aim of this study was to determine the reality of CKD diagnosis and referral practices in Japanese PCCs, whose PCPs are often non-specialists. Thirdly, the average age of the patients was 77 ± 10 years, indicating a high number of elderly individuals. There are few published cohorts of such advanced age, and this study accurately represents Japan's super-aged society. Lastly, despite the different background specialties of PCPs, this study shows that a significant number of clinics have established medical systems using a common platform, enabling the compilation of data in the JAMDAS database. However, given that it was not a random sample of clinics in Japan, and that there may be different characteristics between clinics that use an electronic health record system and those that do not, the generalizability of the results shown here is potentially limited [21].

The study has several limitations. A CKD diagnosis was determined based on CKD-related disease codes. Physicians may have recognized CKD but only provided a diagnosis code when necessary to order prescriptions or examinations. Referral rates were based on the presence of the “fee for providing medical information” code in the billing data. This included referrals to nephrologists for CKD check-ups, and to other medical facilities for different conditions, potentially resulting in overcounting nephrology referrals. However, referral rates were still low in this study. The low referral rate seen in this study may also be affected by patient factors, and not only physician recognition of the importance of CKD. For example, patients with earlier stages of CKD may be asymptomatic and therefore may not be fully aware that they have CKD despite being informed by their physician [31]. Additionally, it is well documented that patients have to psychologically adapt to the different stages of CKD and that patients often struggle to accept the severity of their disease and its progression [32–34]. It is a plausible scenario in clinical practice that a physician may delay referral to a nephrologist or formal CKD diagnosis while the patient is processing this information. Since this study does not clarify the specific processes involved in CKD diagnosis and referrals to nephrologists, further research is needed to validate its findings. The database also lacks medical history from other institutions, which may have led to an underestimation of CKD diagnoses. In addition, CKD patients often use multiple medical institutions for regular examinations and treatment. Finally, inaccurate outcome estimations were possible if single individuals visited multiple medical institutions or received prescriptions from other institutions, which may explain why the absence rate of CKD diagnosis codes in individuals with stages G4 and G5 is higher than previously reported [16], as patients with advanced CKD could already be under the care of specialists and may visit PCPs for other comorbidities. Given these circumstances, PCPs may not need to assign CKD-related diagnosis codes to these patients unless they are managing their CKD.

Overall, this real-world evidence study highlights that CKD-related disease codes are underused by PCPs and CKD is under referred in Japanese clinics. Actions should be taken to increase detection and diagnosis of CKD according to Japanese CKD guidelines.

## Supplementary Information

Below is the link to the electronic supplementary material.

Supplementary file1 (XLSX 205 KB)

Supplementary file2 (DOCX 535 KB)

## Data Availability

To ensure independent interpretation of clinical study results and enable authors to fulfill their role and obligations under the ICMJE criteria, Boehringer Ingelheim grants all external authors access to clinical study data pertinent to the development of the publication. In adherence with the Boehringer Ingelheim Policy on Transparency and Publication of Clinical Study Data, scientific and medical researchers can request access to clinical study data when it becomes available on Vivli—Center for Global Clinical Research Data, https://vivli.org/, and earliest after publication of the primary manuscript in a peer-reviewed journal, regulatory activities are complete, and other criteria are met. Please visit Medical & Clinical Trials | Clinical Research | MyStudyWindow for further information. https://www.mystudywindow.com/msw/datasharing
