# Supplementary material for: First-time diagnosis and referral practices for individuals with CKD by primary care physicians: a study of electronic medical records across multiple clinics in Japan
Source: Clin Exp Nephrol. 2025 May 16;29(10):1342–53. doi: 10.1007/s10157-025-02695-8 (PMC12464027; doi:10.1007/s10157-025-02695-8)
Supplement: Supplementary file 2 — Supplementary file2 (DOCX 535 KB) [file 10157_2025_2695_MOESM2_ESM.docx]

**First-time diagnosis and referral practices for individuals with CKD by primary care physicians: A study of electronic medical records across multiple clinics in Japan**

Haruhito A. Uchida^1,2^, Jun Wada^1^, Yuji Nagao^3^, Katsuhito Ihara^3^

^1^Department of Nephrology, Rheumatology, Endocrinology and Metabolism, Okayama University Faculty of Medicine, Dentistry and Pharmaceutical Sciences, 2-5-1 Shikata-Cho, Kita‑Ku, Okayama, 700-8558, Japan; ^2^Department of Chronic Kidney Disease and Cardiovascular Disease, Okayama University Faculty of Medicine, Dentistry, and Pharmaceutical Science, 2‑5‑1 Shikata-Cho, Kita-Ku, Okayama, Japan; and ^3^Medicine Division, Nippon Boehringer Ingelheim Co., Ltd., 2-1-1 Osaki, Shinagawa-ku, Tokyo, 141-6017, Japan

**Correspondence:** Yuji Nagao or Katsuhito Ihara, Medicine Division, Nippon Boehringer Ingelheim Co., Ltd., 2-1-1 Osaki, Shinagawa-ku, Tokyo, 141-6017, Japan. E-mail: [yuji.nagao@boehringer-ingelheim.com](mailto:yuji.nagao@boehringer-ingelheim.com) or [katsuhito.ihara@boehringer-ingelheim.com](mailto:katsuhito.ihara@boehringer-ingelheim.com)

**Online Resource Supplementary Methods**

**Online Resource 1. Definition of kidney-related laboratory tests**

| **Test** | **JAMDAS laboratory test code** |
| --- | --- |
| Serum creatinine | 160_1 |
| Urine protein | 72_3 |
| Urine albumin | 194_3 |
| Urine creatinine | 245_2 |
| UPCR | 77_1 |
| UACR | 193_1 |
| Dipstick proteinuria | 72_4 |
| Occult blood | 271_1 |
| Urinary sediments (red blood cell) | 272_1 |

*JAMDAS* Japan Medical Data Survey, *UACR* urine albumin-to-creatinine ratio, *UPCR* urine protein-to-creatinine ratio.

**Online Resource 2. Pre-specified CKD-related diagnosis codes**

| **Disease** | ***ICD-10* code** |
| --- | --- |
| CKD, stage I | N18.1 |
| CKD, stage II | N18.2 |
| CKD, stage III | N18.3 |
| CKD, stage IV | N18.4 |
| CKD, stage V | N18.5 |
| CKD, unspecified | N18.9 |
| Hypertensive CKD | I12.0, I12.9, I13.0, I13.1, I13.2 |
| Diabetes with renal manifestation | E10.2, E11.2, E12.2, E13.2, E14.2 |
| Disorders from impaired renal function | N25.0, N25.1, N25.8, N25.9 |

*CKD* chronic kidney disease, *ICD-10* *International Classification of Diseases, Tenth Revision*.

**Online Resource 3. Pre-specified codes for kidney transplant therapies**

| **Name** | **Code** |
| --- | --- |
| Dialysis | *ICD-10* code (Z49.1, Z49.2, Z99.2)  Claim billing code (113002510, 113013610, 114009310, 140036710, 140051010, 140051110, 140052810, 140052970, 140057810, 140057910, 140058010, 140058210, 140058310, 140058410, 140058510, 140058610, 140058970, 140059170, 140060210, 140060310, 140060410, 140062870) |
| Kidney transplant | *ICD-10* code (T86.1, Z94.0) |

*ICD-10* *International Classification of Diseases, Tenth Revision*.

**Online Resource 4.** **Definition of laboratory values meeting the diagnosis criteria according to Japanese CKD guidelines**

Laboratory values meeting the diagnosis criteria are defined as meeting one or more of the following definitions according to Japanese CKD guidelines.^1^

| **Name** | **Definition** |
| --- | --- |
| GFR category | G3a: eGFR between ≥45–<60 mL/min/1.73 m^2^  G3b: eGFR between 30–<45 mL/min/1.73 m^2^  G4: eGFR between 15–<30 mL/min/1.73 m^2^  G5: eGFR <15 mL/min/1.73 m^2^ |
| Proteinuria and/or albuminuria category | A2: dipstick result (±), UPCR ≥0.15–<0.50 g/gCr,  UACR ≥30–<300 mg/gCr  A3: dipstick result ≥ (+), UPCR ≥0.50 g/gCr, UACR ≥300 mg/gCr |
| Hematuria | Occult blood (+), (2+), (3+), (4+)  Urinary sediments red blood cell ≥5/hpf |

*CKD* chronic kidney disease, *eGFR* estimated glomerular filtration rate, *GFR* glomerular filtration rate, *UACR* urine albumin-to-creatinine ratio, *UPCR* urine protein-to-creatinine ratio.

**Online Resource 5. Definition of laboratory values meeting the referral criteria according to Japanese CKD guidelines**

Laboratory values meeting the referral criteria according to Japanese CKD guidelines are defined as meeting one or more of the following definitions.^2^

| **Definition** |
| --- |
| - GFR category   - G3b: eGFR between 30–<45 mL/min/1.73 m^2^   - G4: eGFR between 15–<30 mL/min/1.73 m^2^   - G5: eGFR <15 mL/min/1.73 m^2^ |
| - Proteinuria and/or albuminuria category   - A3: dipstick result ≥ (+), UPCR ≥0.50 g/gCr, UACR ≥300 mg/gCr |
| - GFR category   - G3a: eGFR between 45–<60 mL/min/1.73 m^2^ - Proteinuria and/or albuminuria category   - A2: dipstick result (±), UPCR ≥0.15–<0.50 g/gCr, UACR ≥30–<300 mg/gCr |
| - GFR category   - G1: eGFR ≥90 mL/min/1.73 m^2^   - G2: eGFR between ≥60–<90 mL/min/1.73 m^2^ - Proteinuria and/or albuminuria category   - A2: dipstick result (±), UPCR ≥0.15–<0.50 g/gCr, UACR ≥30–<300 mg/gCr - Hematuria |
| - GFR category   - G3a: eGFR between ≥45–<60 mL/min/1.73 m^2^ - Proteinuria and/or albuminuria category   - A1: dipstick result (-), UPCR <0.15 g/gCr, UACR <30 mg/gCr - Age <40 years |

*CKD* chronic kidney disease, *eGFR* estimated glomerular filtration rate, *GFR* glomerular filtration rate, *UACR* urine albumin-to-creatinine ratio, *UPCR* urine protein-to-creatinine ratio.

**Online Resource 6. Evidence of the referral to other medical institutions**

| **Name** | **Code** |
| --- | --- |
| Fee for providing medical information | Billing code 180016110 |

**Online Resource 7. Kidney function categories**

| **Category** | **Definition** |
| --- | --- |
| CGA category | 18-class category based on a combination of GFR category (6-class) and albuminuria category (3-class) shown below.  e.g., G1-A1, G2a-A2 |
| GFR category | 6-class category;  G1: eGFR ≥90 mL/min/1.73 m^2^  G2: eGFR between ≥60–<90 mL/min/1.73 m^2^  G3a: eGFR between ≥45–<60 mL/min/1.73 m^2^  G3b: eGFR between ≥30–<45 mL/min/1.73 m^2^  G4: eGFR between ≥15–<30 mL/min/1.73 m^2^  G5: eGFR <15 mL/min/1.73 m^2^  eGFR calculated using the Japanese eGFR equation from the Japanese Society of Nephrology.^3^ |
| Proteinuria and/or albuminuria category | 3-class category;  A1: dipstick result (-), UPCR <0.15 g/gCr, UACR <30 mg/gCr  A2: dipstick result (±), UPCR ≥0.15–<0.50 g/gCr, UACR ≥30–<300 mg/gCr  A3: dipstick result ≥ (+), UPCR ≥0.50 g/gCr, UACR ≥300 mg/gCr |

*CGA* *cause* GFR category and albuminuria category, *eGFR* estimated glomerular filtration rate, *GFR* glomerular filtration rate, *UACR* urine albumin-to-creatinine ratio, *UPCR* urine protein-to-creatinine ratio.

**Online Resource 8. Laboratory test codes**

| **Test** | **JAMDAS laboratory test code** |
| --- | --- |
| HbA1c | 227_3 |
| Hemoglobin | 115_1 |
| Serum albumin | 194_1 |
| Serum calcium | 168_1 |
| eGFR | 59_1 |
| Serum creatinine | 160_1 |
| Urine protein | 72_3 |
| Urine albumin | 194_3 |
| Urine creatinine | 245_2 |
| UPCR | 77_1 |
| UACR | 193_1 |
| High density lipoprotein cholesterol | 223_1 |
| Low density lipoprotein cholesterol | 225_1 |
| Triglyceride | 96_1 |
| Blood urea nitrogen | 74_1 |
| Brain natriuretic peptide | 238_1 |
| N-terminal pro-brain natriuretic peptide | 238_2 |
| C-reactive protein | 234_1 |
| Dipstick proteinuria | 72_4 |
| Occult blood | 271_1 |
| Urinary sediments (red blood cell) | 272_1 |

*eGFR* estimated glomerular filtration rate, *HbA1c* glycated hemoglobin, *JAMDAS* Japan Medical Data Survey, *UACR* urine albumin-to-creatinine ratio, *UPCR* urine protein‑to‑creatinine ratio.

**Online Resource 9. Disease codes**

| **Disease** | ***ICD-10* code** |
| --- | --- |
| Type 1 diabetes | E10.x |
| Type 2 diabetes | E11.x, E14.x |
| Hypertension | I10.x, I11.x, I12.x, I15.x |
| Dyslipidemia | E78.0, E78.1, E78.2, E78.3, E78.4, E78.5 |
| Heart failure | I11.0, I13.0, I13.2, I50.x |
| Coronary artery disease | Myocardial infarction, angina pectoris, I25.8, I25.9 |
| Myocardial infarction | I21.x, I22.x, I23.x, I24.1, I25.2 |
| Angina pectoris | I20.0, I20.9, I25.1, I25.7 |
| Stroke | I60.x, I61.x, I62.x, I63.x, I64 |
| Peripheral vascular disease | I70.x, I71.x, I73.x, I77.1, I79.0, I79.2, K55.1, K55.8, K55.9, Z95.8, Z95.9 |
| Atrial fibrillation | I48.0, I48.1, I48.2, I48.9, I49.0 |

*ICD-10* *International Classification of Diseases, Tenth Revision*.

**Online Resource 10. Drug codes**

| **Name** | **ATC code** |
| --- | --- |
| Active vitamin D drug | A11CC |
| RAS inhibitor | ACE inhibitors, ARBs, aldosterone receptor antagonists, ARNIs |
| ACE inhibitor | C09A, C09B |
| ARB | C09C, C09D |
| Aldosterone receptor antagonist | C03DA, H02AA01 |
| ARNI | C09DX |
| Calcium channel blocker | C07FB, C08C, C08D, C08E, C08G, C09BB, C09DB |
| Beta blocker | C07A, C07B, C07D, C07E, C07F |
| Diuretic | Loop diuretics, thiazide diuretics |
| Loop diuretic | C02L, C03B, C03C, C03D, C03E, C03X, C07C, C08G |
| Thiazide diuretic | C03A, C07BA, C07D, C09BA01, C09BA02, C09BA03, C09BA06, C09BA07, C09BA09, C09BA13, C09BX03, C09DA01, C09DA02, C09DA03, C09DA04, C09DA06, C09DA07, C09DA08, C09DX01, C09DX03, C09DX06, C09DX07, C09DX08, C09XA52, C09XA54 |
| Statin | C10AA |
| DPP-4 inhibitor | A10BH, A10BD21, A10BD24, A10BD19, A10BD09, A10BD12, A10BD07, A10BD08, A10BD10, A10BD11, A10BD13, A10BD18, A10BD22, A10BD25, A10BD27, KEGG drug code (D10897, D11109, D11064) |
| SGLT2 inhibitor | A10BK, A10BD15, A10BD16, A10BD19, A10BD22, A10BD23, A10BD24, A10BD25, A10BD27, KEGG drug code (D09978, D10897, D11064) |
| GLP-1 RA | A10BJ |
| Biguanide | A10BA, A10BD02, A10BD03, A10BD05, A10BD07, A10BD08, A10BD10, A10BD11, A10BD13, A10BD14, A10BD15, A10BD16, A10BD17, A10BD18, A10BD20, A10BD22, A10BD23, A10BD25, A10BD26, A10BD27, KEGG drug code (D11109) |
| Sulfonylurea | A10BB, A10BD04, A10BD06, A10BD02 |
| Other oral glucose-lowering drug | A10BC, A10BD04, A10BD06, A10BD03, A10BD05, A10BD14, A10BD17, A10BD26, A10BF, A10BG, A10BX, KEGG drug code (D10160) |
| Insulin | A10A |

*ACE* angiotensin-converting enzyme, *ARB* angiotensin receptor blocker, *ARNI* angiotensin receptor-neprilysin inhibitor, ATC anatomical therapeutic chemical, DPP-4 dipeptidyl peptidase-4, *GLP-1 RA* glucagon-like peptide-1 receptor agonist, *RAS* renin-angiotensin system, *SGLT2* sodium-glucose co-transporter-2.

**Online Resource 11.** **The pre-specified broader definition of CKD diagnosis codes in the sensitivity analysis**

| **Disease** | **ICD-10 code** |
| --- | --- |
| CKD, stage I | N18.1 |
| CKD, stage II | N18.2 |
| CKD, stage III | N18.3 |
| CKD, stage IV | N18.4 |
| CKD, stage V | N18.5 |
| CKD, unspecified | N18.9 |
| Hypertensive CKD | I12.0, I12.9, I13.0, I13.1, I13.2 |
| Diabetes with renal manifestation | E10.2, E11.2, E12.2, E13.2, E14.2 |
| Disorders from impaired renal function | N19, N25.0, N25.1, N25.8, N25.9, N28.9 |
| Nephrotic syndrome | N03.x, N04.x, N05.x |
| Isolated proteinuria with specified morphological lesion | N06.x |
| Hereditary nephropathy | N07.x |
| Glomerular disorders in diseases | N02.x, N08.x |
| Tubulo-interstitial nephritis | N11.x, N12 |

*CKD* chronic kidney disease, *ICD-10* *International Classification of Diseases, Tenth Revision*.

**Online Resource 12**

**Evaluation period for the covariates**

| **Population** | **Reference date** |
| --- | --- |
| Eligible individuals | Day 0 |
| Individuals who met the CKD diagnosis criteria | Date of meeting the CKD diagnosis criteria |
| Individuals who met the CKD referral criteria | Date of meeting the referral criteria |
| Individuals with a CKD diagnosis code | Date of receiving CKD-related diagnosis codes |

For the following covariates, allowances from the reference date were set:

1. **Body mass index (BMI)**
   - BMI was calculated using height and body weight. Height was used for the compilation of existing data regardless of the time of assessment. Body weight was tabulated from 1 year before Day 0 to the reference date of each evaluation period.
   - If multiple data were present for the same evaluation period, the data closest to the reference date were used.
   - If more than one test was recorded on the same day, the minimum value of height and weight was used.
2. **Systolic blood pressure (SBP), diastolic blood pressure (DBP), glycated hemoglobin (HbA1c), hemoglobin (Hb), serum albumin, and serum calcium**

- Evaluation period: reference date ± 90 days.
- If multiple data were present for the same evaluation period, the data closest to the reference date were used.
- If multiple data were present for multiple days during the same duration from the reference date, the data before the reference date were used.
- When more than one laboratory value was recorded on the same day, the maximum laboratory value of SBP, DBP, and HbA1c was used, and the minimum laboratory value of Hb, serum albumin, and serum calcium was used.

1. **Estimated glomerular filtration rate (eGFR), urine albumin-to-creatinine ratio (UACR), urine protein-to-creatinine ratio (UPCR), and qualitative urinary proteinuria**

- Evaluation period: reference date ± 30 days.
- If multiple data exist at the same evaluation period, the data closest to the reference date were used.
- If multiple data exist on multiple days during the same duration from the reference date, the data before the reference date were used.
- If more than one laboratory value was recorded on the same day, the minimum eGFR value and maximum UACR/UPCR value was used.

1. **Comorbidities**

- Medical history/comorbidities from 1 year prior to Day 0 to individual assessment timepoints were summarized.

1. **Medications**

- Evaluation period: reference date – 90 days to the day before the reference date.
- To determine whether or not the drug was administered using prescriptions during the evaluation period.

**Supplementary Results**

**Online Resource 13. Breakdown of CKD diagnosis criteria in individuals who met the CKD diagnosis criteria**

See Supplementary Excel file.

**Online Resource 14. Demographics and characteristics of individuals who met the CKD diagnosis criteria at the time of meeting the criteria stratified by clinic location**

See Supplementary Excel file.

**Online Resource 15.** **Individuals who met the CKD diagnosis criteria at the time of meeting the criteria stratified by CGA category**

Data are numbers of individuals without registered disease codes/individuals who met the CKD diagnosis criteria in each category. Only individuals with data for both the GFR and albuminuria categories were included. Note that if individuals had multiple data for proteinuria and albuminuria categorization during the same duration from the reference date, these data were used to classify the baseline categories in the order of UACR, UPCR, and dipstick result, although all of these data were used to determine whether the criteria were met. This rule of data adoption priority could result in some individuals being assigned to the green (low risk) categories.
*CGA* *cause* GFR category and albuminuria category, *CKD* chronic kidney disease, *GFR* glomerular filtration rate, *UACR* urine albumin-to-creatinine ratio, *UPCR* urine protein-to-creatinine ratio.

**Online Resource 16.** **Demographics and characteristics of individuals at the time of receiving CKD diagnosis codes**

|  | **At the time of receiving CKD diagnosis codes (*n=*28,149)** |
| --- | --- |
| **Age, years** | 69.1 ± 15.8 |
| **Female** | 12,532 (44.5) |
| **BMI, kg/m^2^** | *n*=8,823; 24.7 ± 4.2 |
| <18.5 | 479 (1.7) |
| ≥18.5–25 | 4,555 (16.2) |
| ≥25–30 | 2,790 (9.9) |
| ≥30 | 999 (3.5) |
| Data not available | 19,326 (68.7) |
| **Systolic blood pressure, mmHg** | *n*=15,870;  134.3 ± 20.1 |
| **Diastolic blood pressure, mmHg** | *n*=15,868;  76.1 ± 13.8 |
| **HbA1c, %** | *n*=16,516;  6.3 ± 1.3 |
| **Hb, g/dL** | *n*=20,925;  13.4 ± 2.1 |
| **Serum albumin, g/dL** | *n*=11,200;  4.2 ± 0.5 |
| **Serum calcium, mg/dL** | *n*=5,146;  9.2 ± 0.5 |
| **CKD stage by eGFR, mL/min/1.73 m^2^** | *n*=22,810; 61.4 ± 25.8 |
| G1 (≥90) | 2,840 (10.1) |
| G2 (≥60–90) | 8,273 (29.4) |
| G3a (≥45–60) | 5,584 (19.8) |
| G3b (≥30–45) | 3,823 (13.6) |
| G4 (≥15–30) | 1,809 (6.4) |
| G5 (<15) | 481 (1.7) |
| Data not available | 5,339 (19.0) |
| **UPCR, g/gCr**  Median (Q1, Q3) | *n*=1,488;  0.12 (0.06, 0.37) |
| A1 (<0.15) | 842 (3.0) |
| A2 (≥0.15–0.50) | 329 (1.2) |
| A3 (≥0.15) | 317 (1.1) |
| Data not available | 26,661 (94.7) |
| **UACR, mg/gCr**  Median (Q1, Q3) | *n*=4,964;  23.5 (8.5, 66.3) |
| A1 (<30) | 2,695 (9.6) |
| A2 (≥30–300) | 1,928 (6.8) |
| A3 (≥300) | 341 (1.2) |
| Data not available | 23,185 (82.4) |
| **Urinalysis (qualitative)** | *n*=2,218 |
| A1 (-) | 1,412 (5.0) |
| A2 (±) | 386 (1.4) |
| A3 (+ ~ 4+) | 420 (1.5) |
| Data not available | 25,931 (92.1) |
| **Comorbidities** |  |
| Type 1 diabetes | 337 (1.2) |
| Type 2 diabetes | 18,533 (65.8) |
| Hypertension | 17,792 (63.2) |
| Dyslipidemia | 16,967 (60.3) |
| Heart failure | 6,529 (23.2) |
| Chronic artery disease | 3,880 (13.8) |
| Myocardial infarction | 862 (3.1) |
| Angina pectoris | 3,088 (11.0) |
| Stroke | 1,182 (4.2) |
| Peripheral vascular disease | 2,344 (8.3) |
| Atrial fibrillation | 1,937 (6.9) |
| **Medications** |  |
| Active vitamin D drug | 1,135 (4.0) |
| RAS inhibitor | 9,590 (34.1) |
| Beta-blocker | 2,643 (9.4) |
| Calcium channel blocker | 9,172 (32.6) |
| Diuretic | 3,480 (12.4) |
| Statin | 7,186 (25.5) |
| DPP-4 inhibitor | 6,799 (24.2) |
| SGLT2 inhibitor | 4,921 (17.5) |
| GLP-1 RA | 765 (2.7) |
| Biguanide | 3,885 (13.8) |
| Sulfonylurea | 1,203 (4.3) |
| Other oral glucose-lowering drug | 1,530 (5.4) |
| Insulin | 1,282 (4.6) |

Data are n (%) or mean ± SD unless otherwise indicated.
The evaluation period for the covariates is described in the Supplementary Index.
*BMI* body mass index, *CKD* chronic kidney disease, *DPP-4* dipeptidyl peptidase-4, *eGFR* estimated glomerular filtration rate, *GLP-1 RA* glucagon-like peptide-1 receptor agonist, *Hb* hemoglobin, *HbA1c* glycated hemoglobin, *Q1* first quartile, *Q3* third quartile, *RAS* renin‑angiotensin system, *SD* standard deviation, *SGLT2* sodium-glucose co-transporter-2, *UACR* urine albumin-to-creatinine ratio, *UPCR* urine protein-to-creatinine ratio.

**Online Resource 17. Demographics and characteristics of individuals who met the CKD referral criteria and individuals with or without referral at the time of meeting the criteria**

|  | **Individuals who met the CKD referral criteria** | | | |
| --- | --- | --- | --- | --- |
|  | **Total**  **(*n*=121,335)** | **With referral^a^  (*n*=12,478)** | **Without referral (*n*=108,857)** | **p-value** |
| **Age, years** | 78.2 ± 13.0 | 78.6 ± 13.2 | 78.2 ± 13.0 | <0.01 |
| **Female** | 68,303 (56.3) | 6,554 (52.5) | 61,749 (56.7) | <0.01 |
| **BMI, kg/m^2^** | *n*=22,649; 23.8 ± 4.0 | *n=*2307; 23.6 ± 4.1 | *n=*20 342; 23.8 ± 4.0 | <0.01 |
| <18.5 | 1,727 (1.4) | 232 (1.9) | 1,495 (1.4) | <0.01 |
| ≥18.5–25 | 12,862 (10.6) | 1,280 (10.3) | 11,582 (10.6) |  |
| ≥25–30 | 6,419 (5.3) | 644 (5.2) | 5,775 (5.3) |  |
| ≥30 | 1,641 (1.4) | 151 (1.2) | 1,490 (1.4) |  |
| Data not available | 98,686 (81.3) | 10,171 (81.5) | 88,515 (81.3) |  |
| **Systolic blood pressure, mmHg** | *n=*55,908; 132.8 ± 21.6 | *n=*6,480; 132.4 ± 24.3 | *n=*49,428; 132.8 ± 21.2 | 0.19 |
| **Diastolic blood pressure, mmHg** | *n=*55,891; 73.1 ± 13.7 | *n=*6,473; 73.0 ± 15.4 | *n=*49,418; 73.1 ± 13.5 | 0.73 |
| **HbA1c, %** | *n=*82,984; 6.0 ± 1.0 | *n=*9,046; 6.1 ± 1.2 | *n=*73,938; 6.0 ± 1.0 | <0.01 |
| **Hb, g/dL** | *n=*94,306; 12.5 ± 1.9 | *n=*10,056; 12.1 ± 2.2 | *n=*84,250; 12.6 ± 1.9 | <0.01 |
| **Serum albumin, g/dL** | *n=*42,501; 3.9 ± 0.5 | *n=*4,869; 3.7 ± 0.6 | *n=*37,632; 4.0 ± 0.5 | <0.01 |
| **Serum calcium, mg/dL** | *n=*19,711; 9.1 ± 0.6 | *n=*2,422; 9.0 ± 0.7 | *n=*17,289; 9.1 ± 0.6 | <0.01 |
| **CKD stage by eGFR, mL/min  per 1.73 m^2^** | *n=*113,924; 41.1 ± 12.9 | *n=*11,985; 38.7 ± 14.7 | *n=*101,939; 41.4 ± 12.6 | <0.01 |
| G1 (≥90) | 1,454 (1.2) | 166 (1.3) | 1,288 (1.2) | <0.01 |
| G2 (≥60–90) | 4,656 (3.8) | 504 (4.0) | 4,152 (3.8) |  |
| G3a (≥45–60) | 5,364 (4.4) | 514 (4.1) | 4,850 (4.5) |  |
| G3b (≥30–45) | 89,440 (73.7) | 8,370 (67.1) | 81,070 (74.5) |  |
| G4 (≥15–30) | 11,132 (9.2) | 1,865 (14.9) | 9,267 (8.5) |  |
| G5 (<15) | 1,878 (1.5) | 566 (4.5) | 1,312 (1.2) |  |
| Data not available | 7,411 (6.1) | 493 (4.0) | 6,918 (6.4) |  |
| **UPCR, g/gCr**  Median (Q1, Q3) | *n=*3,250;  0.55 (0.19, 1.03) | *n=*501  0.74 (0.36, 1.78) | *n=*2,749;  0.52 (0.18, 0.97) | <0.01 |
| A1 (<0.15) | 489 (0.4) | 50 (0.4) | 439 (0.4) | <0.01 |
| A2 (≥0.15–0.50) | 932 (0.8) | 95 (0.8) | 837 (0.8) |  |
| A3 (≥0.15) | 1,829 (1.5) | 356 (2.9) | 1,473 (1.4) |  |
| Data not available | 118,085 (97.3) | 11,977 (96.0) | 106,108 (97.5) |  |
| **UACR, mg/gCr**  Median (Q1, Q3) | *n=*2,736;  85.1 (35.5, 395.5) | *n=*249;  131.8 (44.8, 471.6) | *n=*2,487;  81.5 (34.6, 390.3) | <0.01 |
| A1 (<30) | 482 (0.4) | 31 (0.2) | 451 (0.4) | 0.04 |
| A2 (≥30–300) | 1,295 (1.1) | 117 (0.9) | 1,178 (1.1) |  |
| A3 (≥300) | 959 (0.8) | 101 (0.8) | 858 (0.8) |  |
| Data not available | 118,599 (97.7) | 12,229 (98.0) | 106,370 (97.7) |  |
| **Urinalysis (qualitative)** | *n=*18,118 | *n=*1,505 | *n=*16,613 |  |
| A1 (-) | 2,100 (1.7) | 164 (1.3) | 1,936 (1.8) | 0.62 |
| A2 (±) | 2,586 (2.1) | 211 (1.7) | 2,375 (2.2) |  |
| A3 (+ ~ 4+) | 13,432 (11.1) | 1,130 (9.1) | 12,302 (11.3) |  |
| Data not available | 103,217 (85.1) | 10,973 (87.9) | 92,244 (84.7) |  |
| **Comorbidities** |  |  |  |  |
| Type 1 diabetes | 301 (0.2) | 23 (0.2) | 278 (0.3) | 0.13 |
| Type 2 diabetes | 48,764 (40.2) | 4,748 (38.1) | 44,016 (40.4) | <0.01 |
| Hypertension | 78,373 (64.6) | 7,459 (59.8) | 70,914 (65.1) | <0.01 |
| Dyslipidemia | 56,434 (46.5) | 4,740 (38.0) | 51,694 (47.5) | <0.01 |
| Heart failure | 33,697 (27.8) | 3,889 (31.2) | 29,808 (27.4) | <0.01 |
| Chronic artery disease | 20,358 (16.8) | 2,080 (16.7) | 18,278 (16.8) | 0.73 |
| Myocardial infarction | 3,577 (2.9) | 406 (3.3) | 3,171 (2.9) | 0.03 |
| Angina pectoris | 16,794 (13.8) | 1,672 (13.4) | 15,122 (13.9) | 0.13 |
| Stroke | 8,345 (6.9) | 782 (6.3) | 7,563 (6.9) | <0.01 |
| Peripheral vascular disease | 8,765 (7.2) | 854 (6.8) | 7,911 (7.3) | 0.08 |
| Atrial fibrillation | 11,484 (9.5) | 1,267 (10.2) | 10,217 (9.4) | <0.01 |
| **Medications** |  |  |  |  |
| Active vitamin D drug | 7,721 (6.4) | 819 (6.6) | 6,902 (6.3) | 0.33 |
| RAS inhibitor | 41,448 (34.2) | 4,036 (32.3) | 37,412 (34.4) | <0.01 |
| Beta blocker | 12,574 (10.4) | 1,350 (10.8) | 11,224 (10.3) | 0.08 |
| Calcium channel blocker | 39,808 (32.8) | 3,909 (31.3) | 35,899 (33.0) | <0.01 |
| Diuretic | 19,296 (15.9) | 2,175 (17.4) | 17,121 (15.7) | <0.01 |
| Statin | 25,322 (20.9) | 2,192 (17.6) | 23,130 (21.2) | <0.01 |
| DPP-4 inhibitor | 14,383 (11.9) | 1,301 (10.4) | 13,082 (12.0) | <0.01 |
| SGLT2 inhibitor | 9,849 (8.1) | 852 (6.8) | 8,997 (8.3) | <0.01 |
| GLP-1 RA | 908 (0.7) | 80 (0.6) | 828 (0.8) | 0.14 |
| Biguanide | 6,311 (5.2) | 498 (4.0) | 5,813 (5.3) | <0.01 |
| Sulfonylurea | 3,097 (2.6) | 262 (2.1) | 2,835 (2.6) | <0.01 |
| Other oral glucose-lowering drug | 3,642 (3.0) | 320 (2.6) | 3,322 (3.1) | <0.01 |
| Insulin | 1,952 (1.6) | 189 (1.5) | 1,763 (1.6) | 0.38 |

Data are n (%) or mean ± SD unless otherwise indicated.
The evaluation period for the covariates is described in the Supplementary Index.
^a^A CKD referral was defined as the presence of a referral document to another medical institution, regardless of the referral purpose.
*BMI* body mass index, *CKD* chronic kidney disease, *DPP-4* dipeptidyl peptidase-4, *eGFR* estimated glomerular filtration rate, *GLP-1 RA* glucagon-like peptide-1 receptor agonist, *Hb* hemoglobin, *HbA1c* glycated hemoglobin, *Q1* first quartile, *Q3* third quartile, *RAS* renin‑angiotensin system, *SD* standard deviation, *SGLT2* sodium-glucose co-transporter-2, *UACR* urine albumin-to-creatinine ratio, *UPCR* urine protein-to-creatinine ratio.

**Online Resource 18. Individuals who met the CKD referral criteria at the time of meeting the criteria stratified by CGA category**

Data are numbers of unreferred individuals/individuals who met the CKD referral criteria in each category. Only individuals with data for both the GFR and albuminuria categories were included. Note that if individuals had multiple data for proteinuria and albuminuria categorization during the same duration from the reference date, these data were used to classify the baseline categories in the order of UACR, UPCR, and dipstick result, although all of these data were used to determine whether the criteria were met. This rule of data adoption priority could result in some individuals being assigned to the green (low risk) categories.
*CGA cause* GFR category and albuminuria category, *CKD* chronic kidney disease, *GFR* glomerular filtration rate, *UACR* urine albumin-to-creatinine ratio, *UPCR* urine protein-to-creatinine ratio.

**Online Resource Table 19. Demographics and characteristics of individuals who met the CKD referral criteria at the time of meeting the criteria stratified by clinic location**

See Supplementary Excel file.

**Online Resource 20. Proportion of individuals without registered disease codes and unreferred individuals stratified by clinic location**

| **Prefecture** | **Diagnosable individuals** | **Individuals without registered disease codes** | **Proportion of diagnosable individuals without registered disease codes** | **Referrable individuals** | **Unreferred individuals** | **Proportion of referrable individuals without a referral** |
| --- | --- | --- | --- | --- | --- | --- |
| Hokkaido | 4,664 | 4,601 | 98.6 | 3,149 | 2,829 | 89.8 |
| Aomori | N/A | N/A | N/A | N/A | N/A | N/A |
| Iwate | N/A | N/A | N/A | N/A | N/A | N/A |
| Miyagi | 233 | 230 | 98.7 | 138 | 118 | 85.5 |
| Akita | 137 | 134 | 97.8 | 570 | 520 | 91.2 |
| Yamagata | 179 | 166 | 92.7 | 291 | 263 | 90.4 |
| Fukushima | 733 | 723 | 98.6 | 451 | 391 | 86.7 |
| Ibaraki | 1,898 | 1,818 | 95.8 | 1,131 | 1,031 | 91.2 |
| Tochigi | 1,533 | 1,514 | 98.8 | 1,023 | 915 | 89.4 |
| Gunma | 4,874 | 4,772 | 97.9 | 3,313 | 2,936 | 88.6 |
| Saitama | 7,085 | 6,973 | 98.4 | 4,588 | 4,062 | 88.5 |
| Chiba | 8,286 | 8,178 | 98.7 | 7,212 | 6,610 | 91.7 |
| Tokyo | 22,610 | 22,291 | 98.6 | 15,836 | 14,035 | 88.6 |
| Kanagawa | 15,880 | 15,552 | 97.9 | 10,131 | 9,131 | 90.1 |
| Niigata | 3,681 | 3,634 | 98.7 | 2,386 | 2,194 | 92.0 |
| Toyama | 716 | 709 | 99.0 | 419 | 379 | 90.5 |
| Ishikawa | 940 | 934 | 99.4 | 545 | 504 | 92.5 |
| Fukui | 750 | 723 | 96.4 | 415 | 367 | 88.4 |
| Yamanashi | 62 | 60 | 96.8 | 75 | 66 | 88.0 |
| Nagano | 7,191 | 7,080 | 98.5 | 4,468 | 4,042 | 90.5 |
| Gifu | 4,925 | 4,834 | 98.2 | 2,884 | 2,619 | 90.8 |
| Shizuoka | 10,383 | 10,267 | 98.9 | 6,278 | 5,750 | 91.6 |
| Aichi | 19,803 | 19,572 | 98.8 | 11,825 | 10,665 | 90.2 |
| Mie | 7,336 | 7,270 | 99.1 | 4,164 | 3,872 | 93.0 |
| Shiga | 1,065 | 1,061 | 99.6 | 554 | 508 | 91.7 |
| Kyoto | 4,970 | 4,887 | 98.3 | 3,189 | 2,858 | 89.6 |
| Osaka | 12,253 | 12,003 | 98.0 | 7,881 | 6,871 | 87.2 |
| Hyogo | 7,284 | 7,191 | 98.7 | 4,878 | 4,220 | 86.5 |
| Nara | 2,930 | 2,893 | 98.7 | 1,860 | 1,704 | 91.6 |
| Wakayama | 1,379 | 1,342 | 97.3 | 939 | 819 | 87.2 |
| Tottori | N/A | N/A | N/A | N/A | N/A | N/A |
| Shimane | 221 | 219 | 99.1 | 311 | 285 | 91.6 |
| Okayama | 479 | 472 | 98.5 | 396 | 320 | 80.8 |
| Hiroshima | 10,323 | 10,172 | 98.5 | 6,317 | 5,662 | 89.6 |
| Yamaguchi | 1,265 | 1,240 | 98.0 | 981 | 889 | 90.6 |
| Tokushima | N/A | N/A | N/A | N/A | N/A | N/A |
| Kagawa | 236 | 232 | 98.3 | 214 | 187 | 87.4 |
| Ehime | 164 | 164 | 100 | 88 | 79 | 89.8 |
| Kochi | N/A | N/A | N/A | N/A | N/A | N/A |
| Fukuoka | 8,714 | 8,582 | 98.5 | 7,167 | 6,443 | 89.9 |
| Saga | 664 | 653 | 98.3 | 395 | 354 | 89.6 |
| Nagasaki | 2,990 | 2,931 | 98.0 | 2,006 | 1,752 | 87.3 |
| Kumamoto | 1,627 | 1,583 | 97.3 | 939 | 849 | 90.4 |
| Oita | 668 | 656 | 98.2 | 481 | 438 | 91.1 |
| Miyazaki | 589 | 584 | 99.2 | 264 | 249 | 94.3 |
| Kagoshima | 296 | 293 | 99.0 | 307 | 264 | 86.0 |
| Okinawa | 1,383 | 1,357 | 98.1 | 796 | 743 | 93.3 |

"N/A" indicates prefectures with less than 50 individuals who met the CKD diagnosis/referral criteria.
*CKD* chronic kidney disease, *N/A* not applicable.

**Online Resource 21. Breakdown of CKD diagnosis codes in individuals with registered disease codes within 90 days of meeting the CKD diagnosis criteria**

See Supplementary Excel file.

**Online Resource Table 22. Breakdown of CKD diagnosis codes in individuals with registered disease codes within 90 days of meeting the CKD diagnosis criteria (sensitivity analysis)**

See Supplementary Excel file.

**Online Resource 23.** Analysis of the primary outcome based on a broader definition for CKD diagnosis codes (see **Online Resource 12**) **(A)** and the eligible individuals who met the CKD diagnosis criteria at least once **(B)**. *CKD* chronic kidney disease, *EoFU* end of follow-up.

**A**

**
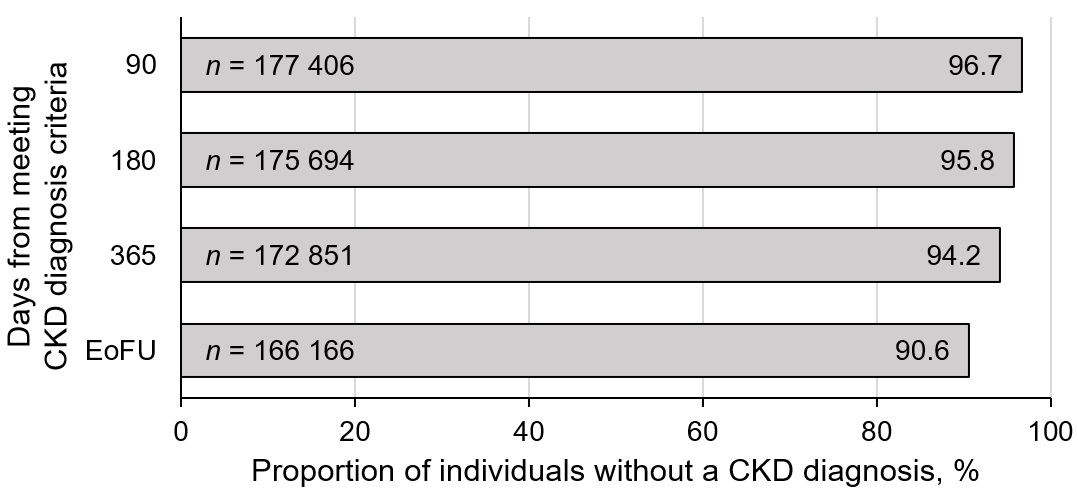
**

Proportion of individuals without registered CKD-related disease codes, %

**B**


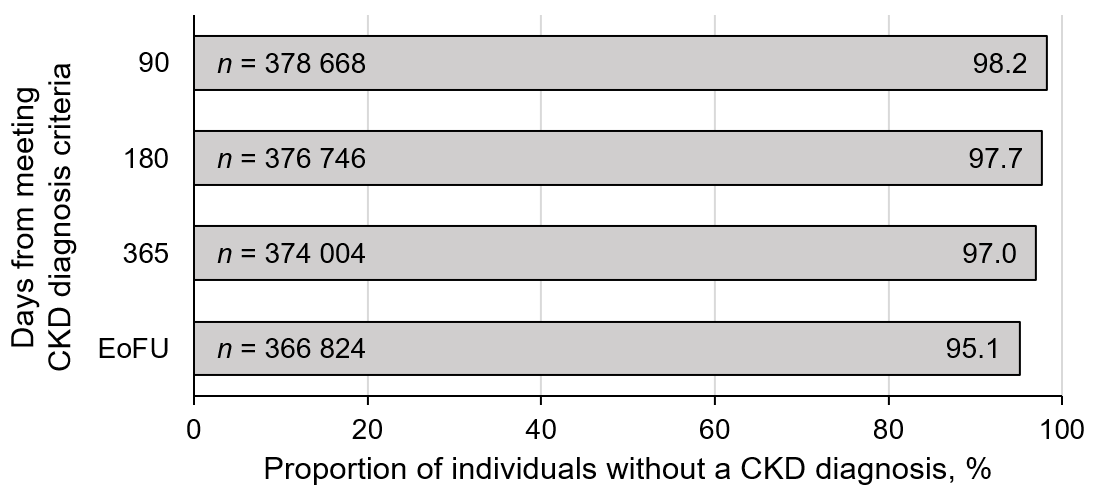


Proportion of individuals without registered CKD-related disease codes, %

**Online Resource 24.** Time gaps from the date of laboratory values meeting the CKD diagnosis criteria to that of receiving CKD-related disease codes using Kaplan-Meier curves for all individuals who met the diagnosis criteria stratified by urinalysis status for those with CKD stage G1 (A), G2 (B), G3a (C), G3b (D), G4 (E), and G5 (F).


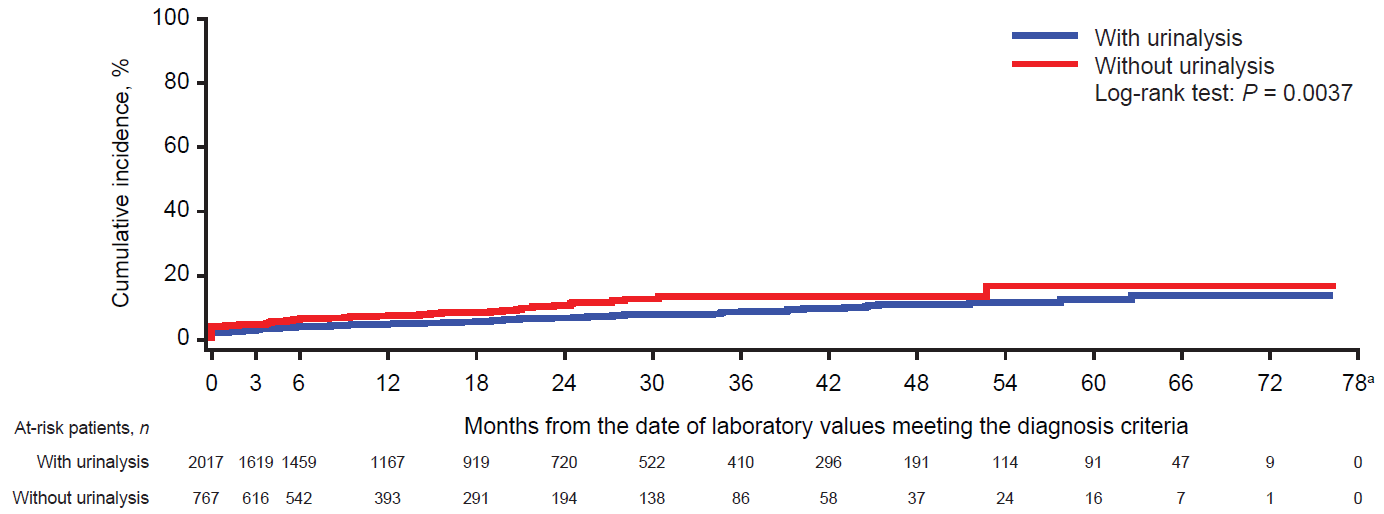

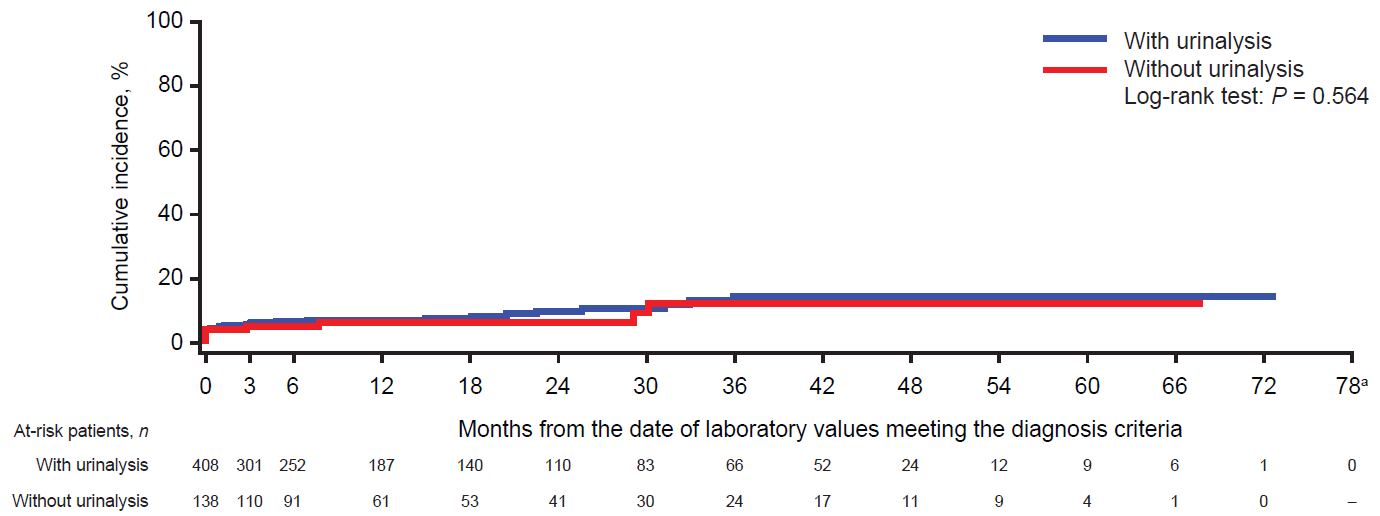
**A**

**B**


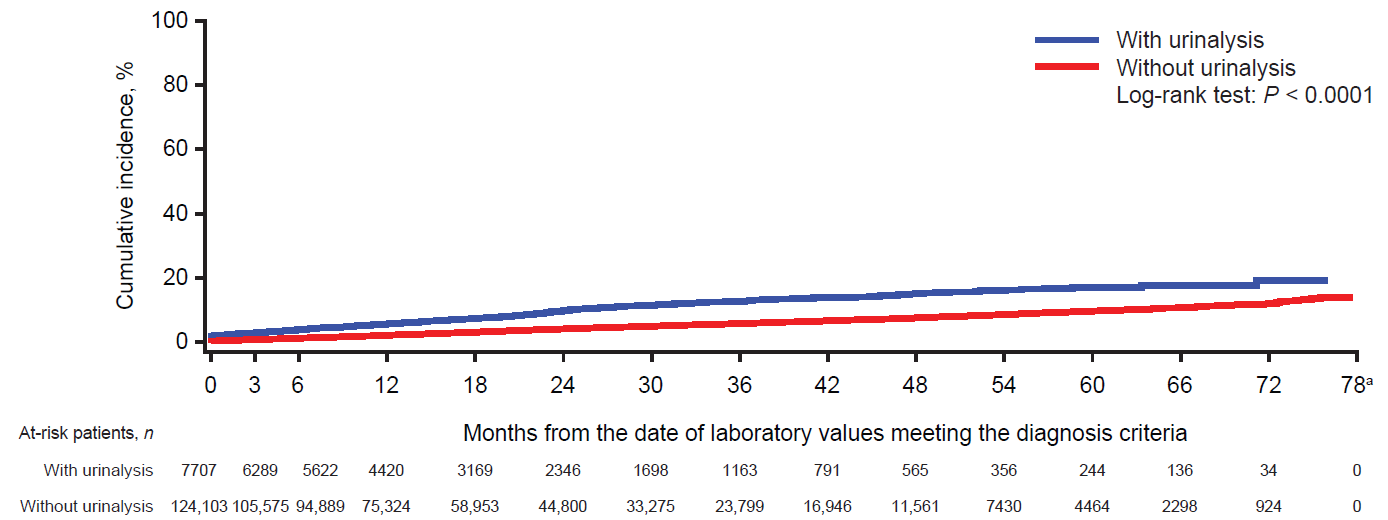
**C**

**D**
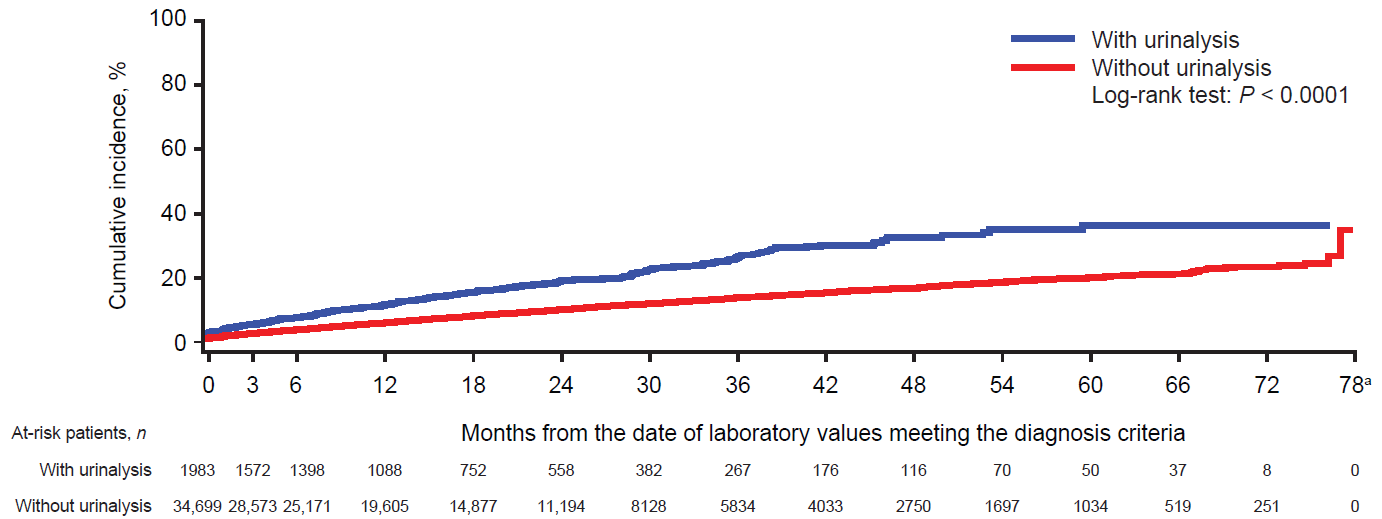


**E**
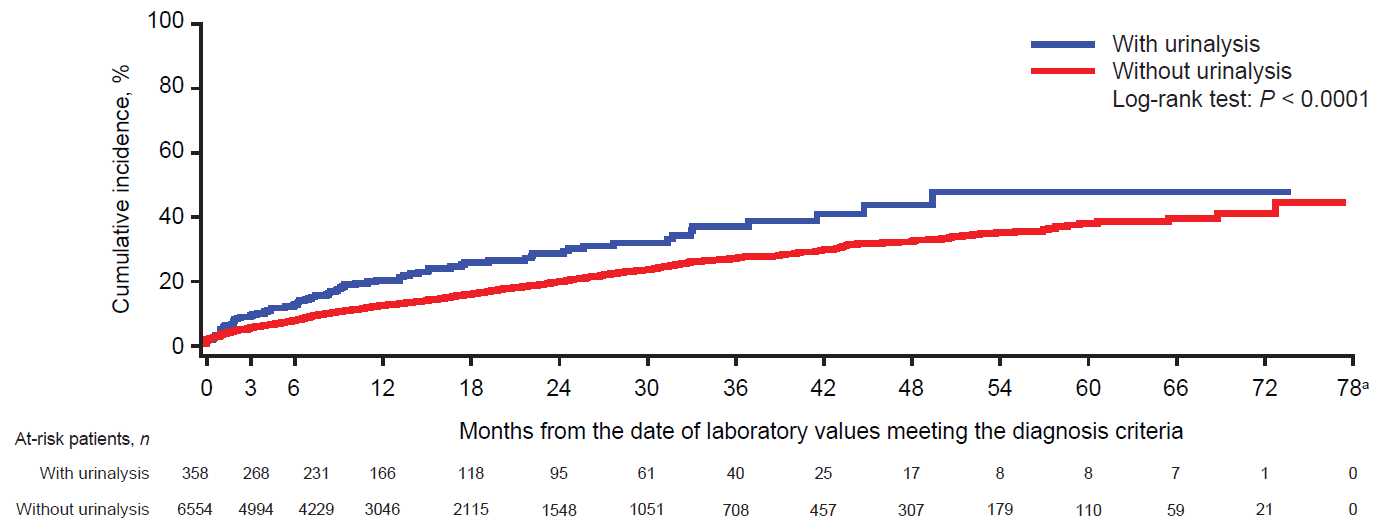


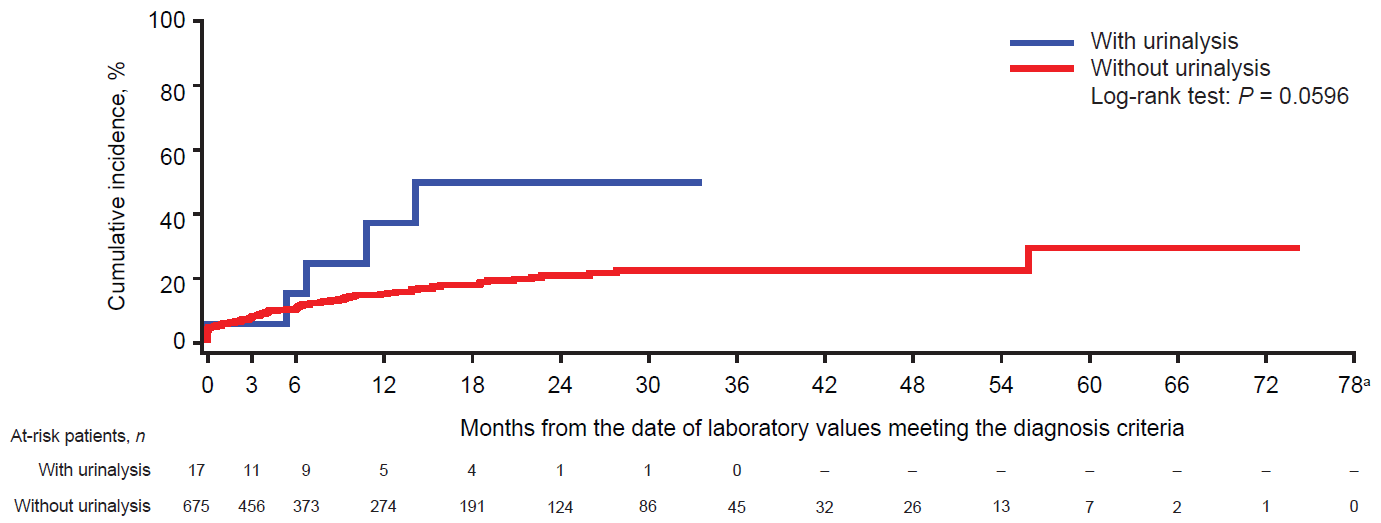
**F**

*CKD* chronic kidney disease.
^a^All individuals were censored at 78 weeks, as this was the maximum follow-up period. This period of 78 weeks was determined by a combination of the data extraction period and the date when laboratory values met the diagnosis criteria.

**References**

1. Japanese Society of Nephrology Guideline. <https://jsn.or.jp/en/guideline/guideline.php>. Accessed March 28, 2024.

2. Fukui A, Yokoo T, Nangaku M, Kashihara N. New measures against chronic kidney diseases in Japan since 2018. *Clin Exp Nephrol.* 2019;23(11):1263-1271.

3. Matsuo S, Imai E, Horio M, et al. Revised equations for estimated GFR from serum creatinine in Japan. *Am J Kidney Dis.* 2009;53(6):982-992.
